# Metastatic renal cell carcinoma presenting as chronic bleeding from the stomach: a rare case report

**DOI:** 10.1093/jscr/rjac045

**Published:** 2022-02-22

**Authors:** Brandon Tapasak, Aron Mcguirt

**Affiliations:** University of Central Florida, College of Medicine, Orlando, FL, USA; University of Central Florida, College of Medicine, Orlando, FL, USA; Bay Pines VA Healthcare System, FL, USA

**Keywords:** case report, renal cell carcinoma, gastrointestinal bleed, gastrectomy, palliative, stomach metastasis

## Abstract

Renal cell carcinoma (RCC) most commonly metastasizes to the lung, adrenals, brain and pancreas, but metastasis to the stomach is uncommon. We present a 77-year-old male who underwent left nephrectomy 9 years previously for RCC with known metastatic disease to the lungs, diaphragm and stomach, and required multiple transfusions for acute blood loss anemia. A previous esophagogastroduodenoscopy revealed a large, friable, ulcerated mass at the gastric cardia. Biopsies of the mass demonstrated clear-cell carcinoma compatible with metastatic RCC. After multiple attempts at endoscopic, procedures and embolization were unsuccessful at controlling bleeding, the patient was treated with palliative total gastrectomy with Roux-en-Y gastric bypass. At discharge, the patient had been hemodynamically stable and tolerating a liquid diet. This case report highlights the presenting symptomology of RCC, explores the rarity of gastric metastases, and reviews current literature on management strategies for these patients.

## INTRODUCTION

In 2021, there were over 76 000 new cases and over 13 000 deaths resulting from kidney and renal pelvis cancer in the USA. The probability of developing kidney and renal pelvis cancer in a male is 1 in 46 from birth to death [1]. Recent data show clear cell renal cell carcinoma (RCC) being the most common subtype of RCC, with a median metastasis of two. The most common sites of metastasis are the lungs, adrenals, brain and pancreas [2]. Paraneoplastic syndromes are the first sign of metastatic RCC in 20% of cases with only 15% of cases showing the classic triad of flank pain, gross hematuria and palpable abdominal mass as most present with signs and symptoms related to metastases [3]. Despite the tendency to metastasize, metastasis of RCC to the stomach is quite rare. There are few reports of gastric metastases with presentations varying between melena, ulceration and blood loss anemia [4, 5]. In these patients, treatment and outcomes are highly variable as well. We report a case of a patient with metastatic clear cell RCC to the stomach causing chronic blood loss anemia and make the case for palliative total gastrectomy as an option for these patients.

## CASE REPORT

**Figure 1:**
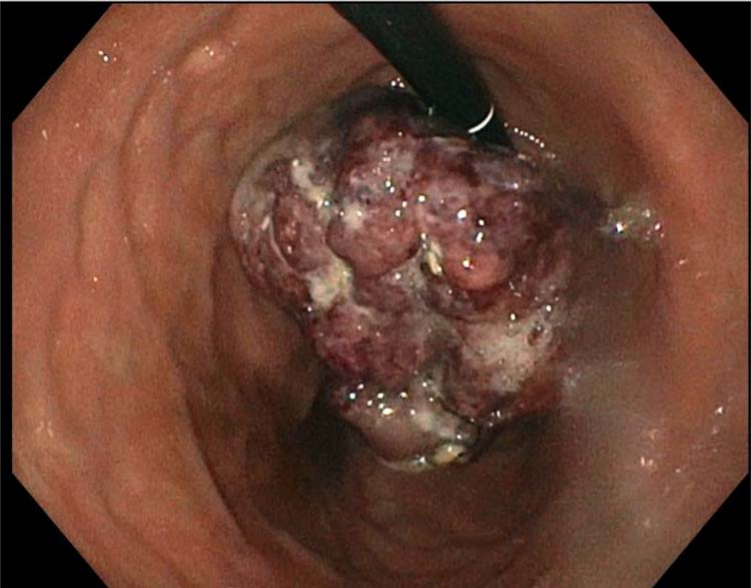
EGD showing stomach mass.

A 77-year-old male was seen in the general surgery clinic in November 2021 for significant gastrointestinal bleeding secondary to metastatic renal cell cancer to the stomach. He was scheduled for a total gastrectomy at that time. His pertinent history consisted of reoccurring blood transfusions from gastric bleed anemia for months most recently requiring a transfusion of one-unit packed RBCs (pRBC) per week. Past medical history was significant for chronic kidney disease stage 3A, hypertension, type 2 diabetes mellitus, hyperlipidemia and stage 4 left kidney clear cell carcinoma with metastases to the diaphragm and stomach. He underwent a left nephrectomy with chemotherapy treatment in April 2016 and open resection of a metastatic lesion to the diaphragm in January 2019. An esophagogastroduodenoscopy (EGD) done in October 2021 showed an 8 cm large, friable, ulcerated mass at the gastric cardia taking up most of the fundus (Fig. 1). Biopsies of the mass demonstrated clear-cell carcinoma compatible with metastatic RCC.

**Figure 2:**
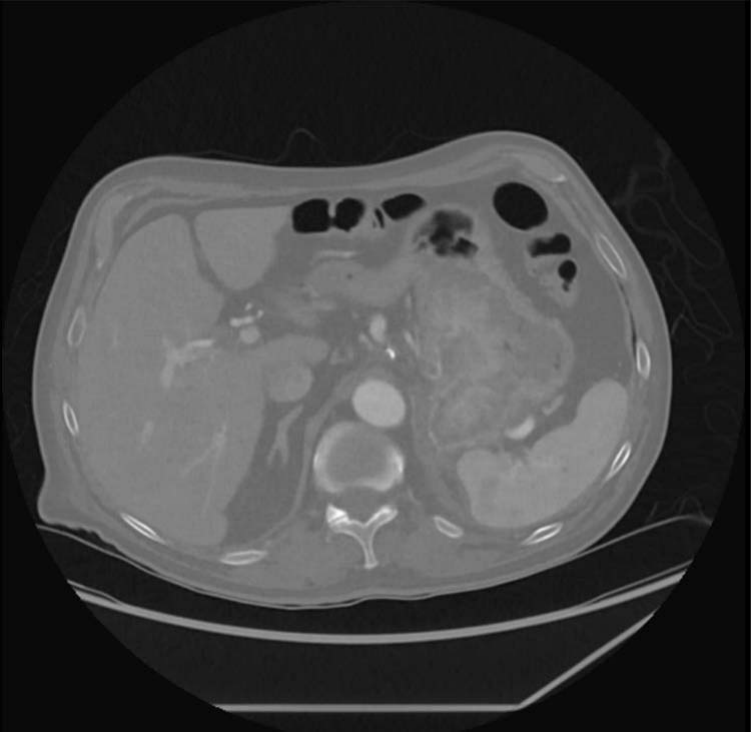
CT abdomen/pelvis with stomach mass.

Prior to his scheduled surgery he presented to the emergency department in December 2021 with complaints of dizziness and syncope. Initial examination showed tachycardia and orthostatic hypotension consistent with hypovolemia. Cardiopulmonary and abdominal exams were unremarkable. Laboratory data were significant for a hemoglobin of 7.5. He was admitted and treated with intravenous fluids and two units of pRBCs. A computed tomography (CT) abdomen/pelvis done on admission showed the stomach mass similar to the previous scan done four months prior (Fig. 2).

**Table 1:** Summary of RCC metastases to stomach and multiple or unspecified sites

Author	Presentation	Treatment	Outcomes
Hakim *et al*. [5]	Melena, hematemesis	Endoscopic, palliative radiation	ND
Orosz *et al*. [6]	Melena, blood loss anemia	Endoscopic	ND
Bernshteyn *et al*. [7]	Melena, dyspnea	Endoscopic	ND
Arakawa *et al*. [8]	Anorexia, pyrexia, malaise	No surgical, Axitinib	ND
Sakurai *et al*. [4]	Melena, anemia	Partial	Died 4 months
Eslick and Kalantar [9]	Melena	Endoscopic	6-year survival
Tiwari *et al*. [10]	Melena, hematemesis, fatigue, abdominal tenderness	Subtotal	Died 2 months
Kibria *et al*. [11]	Melena, fatigue, dizziness	Palliative	Died 2 months
Yamamoto *et al*. [12]	Melena	Partial	Died 1 month
Pollheimer *et al*. [13]	Anemia, epigastric pain	Partial, sunitinib	2-year survival
Pollheimer *et al*. [13]	Upper GI hemorrhage, melena, anemia	Partial	Died 3 months
Pollheimer *et al*. [13]	Melena, anemia	No surgical, interferon	Died 5 months
Pollheimer *et al*. [13]	Asymptomatic (incidental)	No surgical, interferon	Died 4 months
Pollheimer *et al*. [13]	Epigastric pain, nausea, emesis	Tamoxifen, no surgical	Died 19 months

The patient required multiple transfusions (six units over 2 days) for hemoglobins below 7.0 gm/dL while hospitalized. Interventional radiology was consulted to perform embolization of the gastroduodenal and gastroepiploic arteries to obtain vascular control for surgery. This slowed the bleeding allowing for adequate resuscitation. Prior to surgery, hemoglobin was 8.1 gm/dL and one unit of pRBCs was given. The patient underwent total gastrectomy with Roux-en-Y reconstruction and feeding jejunostomy on hospital Day 3. There were no major complications. The patient was transfused with one unit again after surgery. Hemoglobin had stabilized with a range of 8.1–10.1 gm/dL on postoperative Day 4. He underwent a swallow study, which demonstrated no leak and was started on a clear liquid diet on postoperative Day 6. He had a stable hemoglobin and was tolerating a full liquid diet upon discharge.

## DISCUSSION

A small literature review of case reports was performed to determine the presentation, treatment and outcomes of patients with RCC metastases to multiple sites including the stomach (Table 1). Patients with solitary gastric metastases, unspecified metastases and studies in a language other than English were excluded. Literature about gastric metastasis of RCC was realized using the electronic database Medline via PubMed (2008–2021).

Metastases to the stomach are uncommon. Incidence is reported to vary between 0.2 and 0.7%. The highest prevalence of gastric metastases are seen from primary breast cancer (27%) and lung cancer (23%) with 7.6% being from renal cell cancer [6]. The median interval between treatment of RCC primary tumor and diagnosis of metastatic stomach tumor is 50–78 months, whereas metastases from lung cancer and malignant melanoma are detected within 2 years [7]. Since metastatic gastric tumors from breast cancer and RCC are slow-growing, there may be similarities in clinical characteristics.

Clinical presentation of gastric metastases is often asymptomatic or nonspecific unless the metastases invade the gastric mucosa or serosa. Since the stomach is neglected until there is symptomatic anemia or bleeding, metastases are detected at an advanced stage [8].

For solitary gastric metastases, surgical resection is the preferred treatment as this may contribute to long term survival. Furthermore, surgical resection of metastatic gastric tumors may be recommended to control hemorrhaging and result in improved quality of life. However, recent studies have shown that long-term survival in these patients is rare [9]. As further shown by the literature review, the prognosis for this disease is very poor as most of these patients did not live past 2 years.

As supported in the literature, surgical treatment remains the best therapeutic option for a solitary gastric metastasis. Many studies have even shown that median patient survival was higher after surgical treatment of gastric metastasis in the case of solitary and multiple metastatic sites [14]. Palliative gastrectomy should be an option for decreased burden on patients and the healthcare system as was for our patient. As the patient required a transfusion almost every week for his gastric hemorrhage, systemic chemotherapy was not a viable short-term option. Although endoscopic surgery seems to be the standard for more recent cases, this is mostly dependent on the clinical symptoms and contraindications to gastrectomy per patient.
